# A Contemporary Approach to the Treatment of Perioperative Bronchospasm

**DOI:** 10.31480/2330-4871/112

**Published:** 2020-02-10

**Authors:** Christian Bohringer, Daniel Copeland, Hong Liu

**Affiliations:** Department of Anesthesiology and Pain Medicine, University of California Davis Health, Sacramento, California, USA

**Keywords:** Perioperative bronchospasm, Capnography, Beta-2 agonists, Hyperinflation

## Abstract

The incidence of asthma is increasing, and the ageing of the United States population is leading to an increase in the prevalence of patients living with chronic obstructive pulmonary disease. This has led to an increased need to manage bronchospasm in the perioperative period. Very effective methods to treat bronchospasm like intravenous dexmedetomidine, lidocaine, magnesium, ketamine and steroids as well as inhalational sevoflurane are available but are currently underused. Inhaled beta-2 agonists like albuterol are instead often relied upon as the sole therapeutic agent – often with limited response. Just like with pain management, the successful treatment of perioperative bronchospasm requires a multimodal approach. The diagnosis of intraoperative bronchospasm must be rapid, and the treatment must be effective to prevent the dreaded “dynamic hyperinflation syndrome”. This article reviews the diagnosis of bronchospasm and the contemporary treatment methods that should be employed to prevent bronchospasm-related morbidity and mortality during the perioperative period.

## Introduction

Both asthma and chronic obstructive pulmonary disease (COPD) are getting more common [1,2]. This may be related to increased urbanization and air pollution around the world and an ageing population in developed countries. Obesity is also a predisposing factor for the development of asthma [3]. The obesity epidemic in the United States is therefore an additional cause for the increased incidence of bronchospasm that is now being observed in the perioperative period. Asthmatics have also been recognized to have a higher prevalence of sleep apnea symptoms than non-asthmatics, and these symptoms are associated with a higher disease burden [4]. In classical medical teaching a distinction has been drawn between two diseases. The first is asthma which is characterized by reversible and episodic bronchospasm, and usually affects children and younger adults. The second is COPD and typically affects older patients with a history of long-term smoking. However, alpha-1 antitrypsin deficiency can lead to the development of COPD at a young age. Recently an overlap syndrome between these conditions has been described which has been named the asthma-COPD overlap syndrome [5]. This overlap syndrome was found to have a higher incidence in women and younger patients and had a higher mortality than either asthma or COPD alone [5]. Severe COPD may be associated with pulmonary hypertension that can be identified by palpating a right ventricular impulse at the left sternal edge, electrocardiographic changes of right ventricular hypertrophy, and by echocardiography. If pulmonary hypertension is present, the patient and the surgeon need to be informed of the greatly elevated risk of surgery, and invasive monitoring should be considered both during and after the surgery.

## Differential Diagnosis of Bronchospasm

**Figure 1:**
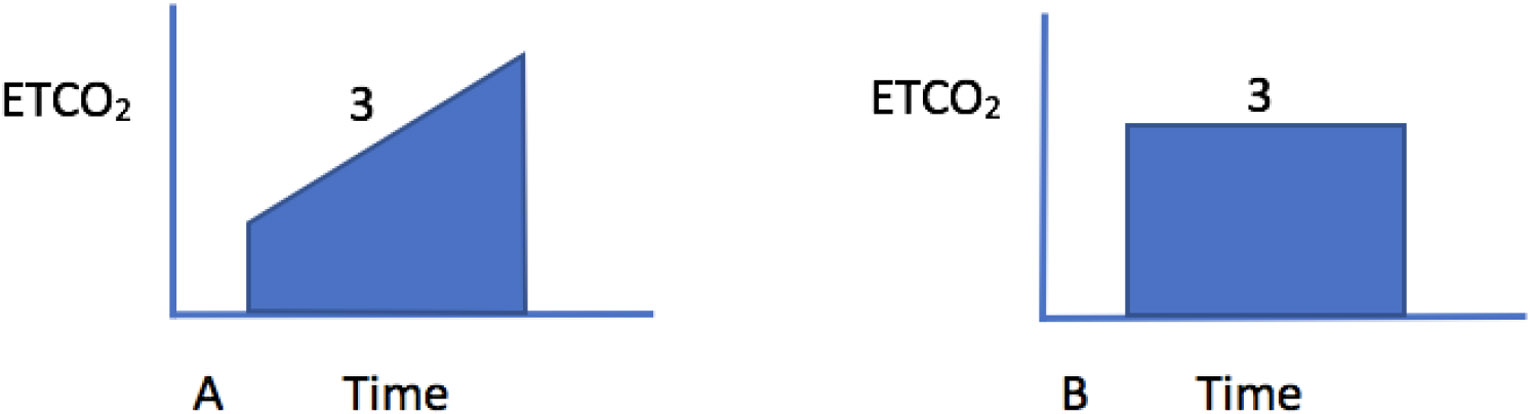
Representative capnograph trace during bronchospasm that there is an upslope during phase 3 (A). In a normal lung, there is usually a plateau during phase 3 (B). The degree of upslope is proportional to the arterial to end-tidal PaCO_2_ gradient and the amount of ventilation-perfusion mismatch that is present.

**Figure 2:**
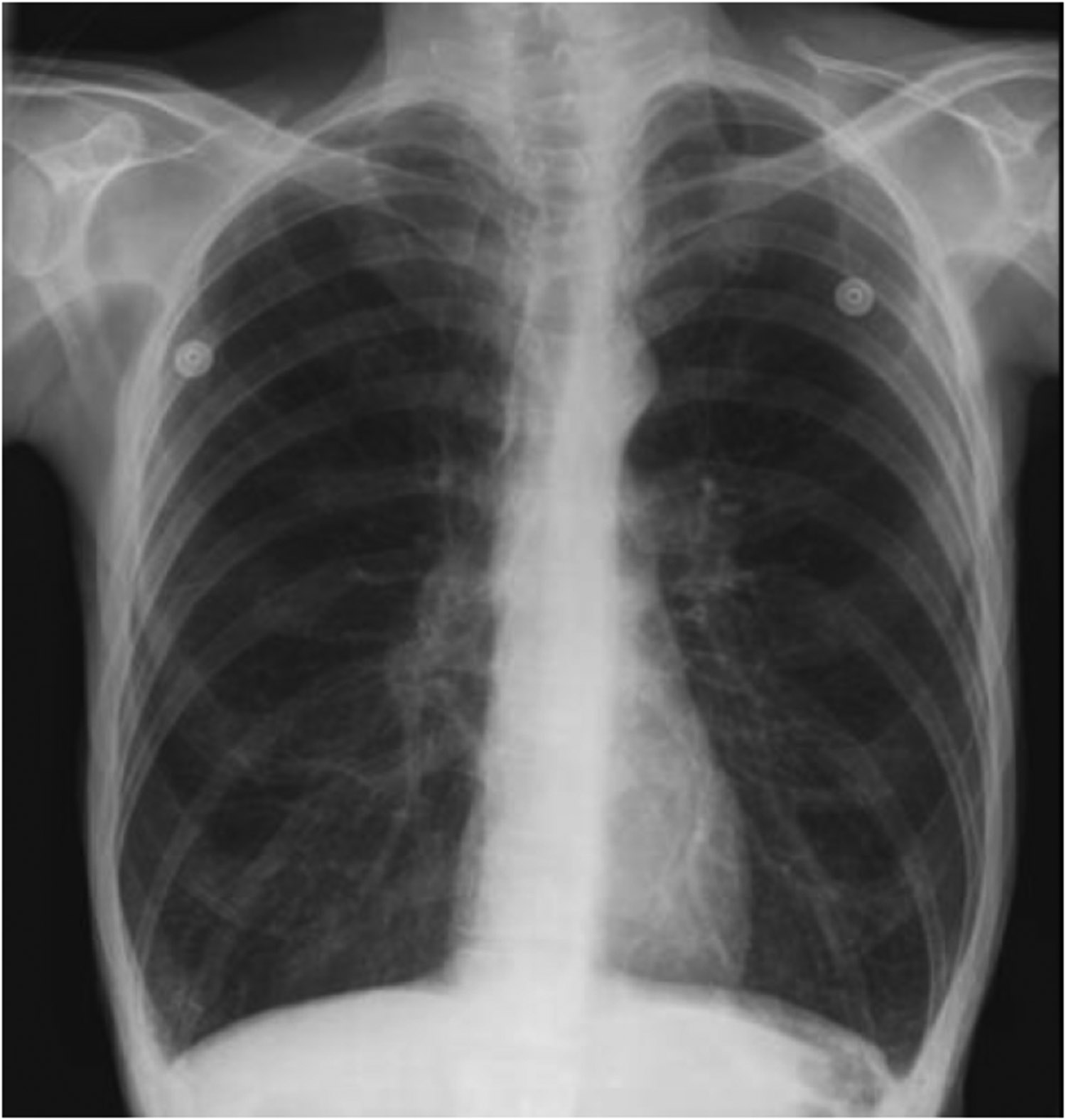
Representative chest X-ray of bronchospasm. During acute bronchospasm there is hyperinflation of the lungs with compression of the heart into a tubular shape and displacement of the diaphragm in a caudal direction. The increase in intrapulmonary pressure may lead to decreased venous return to the heart and a decrease in cardiac output.

Bronchospasm usually presents with audible wheezing that can often be heard even without the aid of a stethoscope. There is a delayed expiration phase and the patient has obvious difficulty expelling the air during exhalation. The chest is hyper-inflated and the jugular venous pressure is elevated. The wheezing is bilateral and desaturation on pulse oximetry is usually not a prominent feature. Shortness of breath in asthma is a very subjective symptom and a peek flow meter should be used to assess the severity of the attack. The peak flow meter readings should be evaluated in comparison to the patient’s usual peak flows at home when he or she is well [6]. If the bronchospasm is severe then the intra-bronchial gas flow is greatly reduced, and the wheezing may be more difficult to identify. A capnography tracing shows an upslope during phase 3 and the absence of an end expiratory plateau (Figure 1). In a patient with the physical exam findings of peripheral edema, auscultation of crackles and a third heart sound, and a low pulse-oximetry saturation, the diagnosis is likely to be pulmonary edema rather than bronchospasm. The chest X-ray will clearly distinguish between these two conditions. With bronchospasm, the lung fields are hyper-expanded and the heart will be compressed into a tubular shape (Figure 2). In contrast, with pulmonary edema the heart is often enlarged and the lung fields are opaque and show a characteristic bat’s wing appearance.

Beta-2 agonist treatments can sometimes help or hinder the diagnostic process. Heart failure may be misdiagnosed as asthma for a significant period of time, because the beta-2 agonist therapy often improves the symptoms of heart failure due to some cross-over beta-1 stimulation of the heart. This apparently successful response to beta-2 agonist therapy will then falsely reaffirm the diagnosis of air flow limitation in the clinician’s mind. In contrast, intrathoracic airway obstruction due to a mass from a lymphoma or a lung cancer compressing the trachea also presents with bilateral wheezing. This usually does not respond to beta-2 agonists. Signs of concomitant superior vena cava obstruction should be looked for in this scenario. If the wheezing is unilateral, then the diagnosis is most likely foreign body aspiration. This condition occurs more often in children and trauma patients [7,8]. Aspirated food particles or a broken tooth are common culprits.

To complicate matters, an intraoperative bronchospasm may be the first sign of aspiration of gastric contents and this diagnostic possibility should be especially considered when a laryngeal mask airway (LMA) is in use. A suction catheter should be placed through the LMA as a diagnostic maneuver to rule out the presence of gastric contents in the trachea. If the patient is intubated with an endotracheal tube (ETT), aspiration past the inflated cuff of the ETT is still possible but far less likely. Repeated suctioning should be avoided unless gastric contents are identified, because this endobronchial stimulation will augment the reflex induced bronchoconstriction. A kinked ETT is often misdiagnosed as intraoperative bronchospasm. It usually presents with high airway pressure and an upslope of the capnography tracing during the plateau phase. Often this ETT kink occurs after turning a patient prone with an ETT *in situ.*

Occasionally severe bronchospasm is the main or even the only clinical manifestation of an anaphylactic reaction. Severe bronchospasm at the beginning of surgery should therefore be investigated with a serum mast cell tryptase blood test to rule out anaphylaxis, especially if this occurs in a patient without a previous history of bronchospasm [9]. If the tryptase is positive, the patient should be referred to an allergy clinic for skin testing. The information gained from this will be very useful during subsequent anesthetics. Fluid overload and pulmonary edema may also be misdiagnosed as bronchospasm during the operation but low saturations on pulse oximetry, frothy pink fluid from the ETT and the chest X-ray (CXR) easily distinguish between these conditions (Table 1).

## Prevention of Perioperative Bronchospasm

When poor control of asthma or COPD is identified in the pre-op clinic, a five-day course of 40 mg/day oral prednisone that is added to the patient’s usual inhaler therapy is effective in preparing the patient for surgery and will make it less likely that the surgery will get cancelled due to bronchospasm on the day of surgery [10,11]. Common precipitants of intraoperative bronchospasm are airway stimulation with endotracheal intubation, inhalation of cold dry gases, endobronchial suctioning and bronchial stimulation with rigid bronchoscopes or flexible intubation scopes. All of these should be avoided in patients at risk of bronchospasm unless they are truly necessary (Table 2).

Spinal and epidural anesthetics do not exacerbate bronchospasm in clinical practice, even though they abolish sympathetic tone and would therefore theoretically be expected to increase bronchial smooth muscle tone. Central neuraxial blocks can be employed safely if there is any concern about bronchospasm, and the surgery can be performed under such a block [12-14]. The use of a regional anesthetic will avoid airway instrumentation and the administration of dry gases that were not humidified by passage through the patient’s nose [15,16]. Regional anesthesia has been shown to be an excellent strategy to prevent perioperative bronchospasm [12].

If a general anesthetic is necessary for the surgery, a LMA is less likely to precipitate bronchospasm than an ETT [17]. The inspired gases should be warmed and humidified as much as possible. The insertion of a heat moisture exchanger into the breathing circuit and the administration of a low fresh gas flow of about 2 liters per minute will help to maintain the inspired gas warm and humidified. When ventilating asthmatics with an intensive care ventilator and high fresh gas flows, a heated water bath humidifier should be used to ensure adequate humidification of inspired gases. Suction of the bronchi should only be performed if truly necessary because it may exacerbate reflex bronchospasm. If aspiration is considered to be a potential cause for the bronchospasm, suctioning can be of diagnostic value. Repeated suctioning and lavage with saline should however be avoided.

Histamine releasing drugs should also be avoided in patients at risk for bronchospasm. Vancomycin, racemic atracurium, morphine, succinylcholine, mivacurium and sodium pentothal all release significant amounts of histamine and cause significant bronchospasm. The muscle relaxant rapacuronium was taken off the market because it caused significant bronchospasm.

The cis-isomer of atracurium is devoid of histamine release and can be safely administered to asthmatics. Iodine based contrast agents should also be avoided in asthmatics due to their significant histamine release (Table 3). Non-ionic lower osmotic contrast agents like the ones based on gadolinium are much safer and release much less histamine [18-20].

Desflurane has very irritant physicochemical properties that reliably produce bronchospasm at higher concentrations and should not be administered to patients at risk of bronchospasm [21].

Ketorolac and other non-steroidal anti-inflammatory drugs should be avoided because their interference with prostaglandin synthesis leads to bronchospasm [22,23]. Non-selective beta blockers like labetalol and propranolol also need to be avoided [24]. Beta-1 selective blockers like metoprolol and atenolol also frequently exacerbate bronchospasm and should only be used if absolutely necessary [25]. The class III antidysrhythmic drug amiodarone also has significant beta blocker (or Class II) antidysrhythmic activity and frequently causes bronchospasm [26]. If beta-2 agonist induced tachydysrhythmias need to be treated, dexmedetomidine is a much better choice than amiodarone or beta-1 blockers because of its powerful bronchodilator properties.

## Treatment of Bronchospasm

### Beta-2 agonists

Inhaled beta-2 agonists like albuterol are the first line therapy in patients that do not yet have an intravenous (IV) access established (Table 4). Disadvantages of beta-2 agonists include tachycardia and patient anxiety due to their sympathomimetic effects. Beta-2 selectivity is only partial and tachy-dysrhythmias due to cross-over stimulation of beta-1 cardiac receptors usually limit the dose of beta-2 agonists that can be safely administered. Beta-2 agonists also frequently induce tremor, hypokalemia, ventilation-perfusion mismatch and a lactic acidosis that need to be carefully monitored [27,28]. When the patient is intubated it is difficult to time the actuation of the metered dose inhaler with respiration. The inhaled drugs also have the tendency to precipitate on the wall of the ETT. At least 8 puffs of a metered dose inhaler should therefore be administered as the initial dose. Metered dose inhalers administered via an in-circuit spacer are more effective at delivery than nebulizers. Vibrating mesh nebulizers have been found to be more effective than jet nebulizers [29]. When the bronchospasm is severe and the minute volume is low, inhaled beta-2 agonists are not very effective, because there is a very limited delivery of the drug to the bronchi. If the inhaled drug does not reach the bronchi, the inhalational therapy will not be able to exert its beneficial effect. Intravenous administration of bronchodilator drugs rather than inhalational therapy should therefore be employed whenever bronchospasm is severe or does not seem to readily respond to inhaled therapy.

### Intravenous dexmedetomidine

Dexmedetomidine is a powerful bronchodilator drug and is very much underused in the treatment of asthma and COPD (Table 4). It completely reverses bronchospasm following a histamine challenge in dogs [30] and it reverses acetylcholine induced bronchoconstriction in guinea pig tracheal mucosa. Three mechanisms for the bronchodilator effect of dexmedetomidine have been identified. Intraoperative bronchospasm in response to endotracheal intubation and instrumentation is mainly mediated via the vagus nerve and the irritant C-fibers. Dexmedetomidine reverses bronchospasm by antagonizing acetylcholine at the post-ganglionic cholinergic nerve ending of the vagus nerve, by producing direct relaxation of bronchial smooth muscle, and by inhibiting substance P release by the C fibers [31]. These three actions are very potent and are responsible for the powerful bronchodilator response of dexmedetomidine. When patients with status asthmaticus in the intensive care unit (ICU), who are on maximal doses of all of the other conventional therapies for asthma, receive dexmedetomidine in doses large enough to decrease the heart rate, the bronchospasm usually disappears and intubation is no longer deemed necessary by the ICU team. In the authors’ experience, many intubations of patients with status asthmaticus in the ICU could be avoided after dexmedetomidine was administered in preparation for intubation. Dexmedetomidine also prevents noxious airway reflexes like laryngospasm and bronchospasm at the end of surgery [32] and has been shown to be superior for this to both lidocaine and fentanyl [33,34]. Dexmedetomidine is also useful because of its anxiolytic effect. Midazolam does not have any bronchodilator effect and dexmedetomidine should, therefore, be used as the pre-operative anxiolytic of choice in any patient who has pre-operative bronchospasm [35]. Dexmedetomidine should be administered in 20 mcg boluses and titrated to the heart rate and level of anxiolysis that is desired. The sedative properties and anti-shivering effects of this drug are desirable, because they counteract the cerebral agitation and the tremor caused by high doses of continuous beta-2 agonist nebulizers. Dexmedetomidine has a long half-life of two hours and it therefore makes sense to administer it by IV bolus doses rather than by continuous IV infusion [36]. Ischemic heart disease (IHD) and COPD are both caused by cigarette smoking and frequently occur together in the same patient [37]. Unlike the beta-2 agonists that usually produce significant tachycardia, dexmedetomidine slows the heart rate. In patients with co-existing IHD it is a safer drug to treat bronchospasm than beta-2 agonists. The negative chronotropic effect of dexmedetomidine is not associated with a negative inotropic effect. It is a much better agent to slow the heart rate than a selective beta-1 blocker like atenolol or metoprolol. Selective beta-1 blockers also frequently exacerbate bronchospasm, and they should be avoided in patients with bronchospasm because the selectivity is only relative and incomplete. Just like most patients will develop tachycardia with a “selective” beta-2 agonist like albuterol, most patients will develop a degree of bronchospasm following the administration of a “selective” beta-1 blocker like atenolol or metoprolol.

### Lidocaine

Intravenous lidocaine is another powerful bronchodilator that is underused for the treatment of bronchospasm in clinical practice. It has a profound effect of reversing acetylcholine induced bronchospasm in guinea pig tracheal mucosa [38]. It also reverses histamine induced bronchospasm in healthy volunteers when given by IV injection or when administered by inhalation. Both lidocaine and its oral analogue mexiletene block reflex induced bronchoconstriction in asthmatic patients [39]. When administered to asthmatic patients after endotracheal intubation it mitigates bronchoconstriction [40]. Nebulized lidocaine has also been shown to reduce bronchospasm during awake flexible scope intubation in asthmatics [41]. In awake volunteers with known airway hyper-reactivity intravenous lidocaine and bupivacaine both reversed acetylcholine induced bronchospasm [42]. Lidocaine is more effective in blocking bronchoconstriction in response to noxious stimuli than allergic bronchoconstriction [43]. This makes it a very useful agent to combat intraoperative bronchospasm which is primarily caused by noxious stimuli that are initiating vagal and c-fiber mediated bronchoconstriction. The mechanism of action extends beyond just providing topical airway anesthesia. The local anesthetic dyclonine produced significantly longer lasting and more intense airway anesthesia, but could not reverse histamine induced bronchospasm in awake volunteers like lidocaine and ropivacaine did [44].

### Magnesium

Intravenous magnesium acts as a direct bronchodilator [45-47] and it appears to be effective in both children and adults [48]. It inhibits calcium uptake in bronchial smooth muscle and prevents it from contracting [49]. Many case reports support its use during severe episodes of bronchospasm [50-52]. It is mainly recommended for patients with acute severe asthma [53]. Large doses of magnesium of 2-4 g are advocated (bolus dose of 25-75 mg/kg over 20 minutes). When administered to awake patients, it needs to be administered slowly over 20 minutes to prevent hot flush and sudden vasodilation. When it is administered intra-operatively one needs to remember that magnesium potentiates the action of neuromuscular blocking agents and their dose needs to be reduced. Particular attention needs to be paid to ensuring that adequate reversal of neuromuscular blockade has been achieved prior to extubating the patient. Large doses of magnesium will also cause vasodilation and may require the administration of vasopressors. Inhaled magnesium does not seem to be effective like IV administration in treating bronchospasm and is no longer recommended [54-56].

### Sevoflurane

Inhaled sevoflurane is known to be a powerful bronchodilator and unlike desflurane, it does not have any irritant physicochemical properties. It has been shown to be as effective as isoflurane in protecting against bronchospasm in dogs [57]. It is a well-accepted rescue therapy for patients with life-threatening bronchospasm [58]. It is beneficial in ICUs because it can be used as a sedative and as a bronchodilator at the same time. It has the added benefit of reducing metabolism and CO_2_ production. This is of great benefit in life-threatening asthma. When the minute ventilation is severely limited, the reduced metabolic rate and CO_2_ caused by sevoflurane production will impede the development of a respiratory acidosis. It is a much more powerful bronchodilator than propofol and was associated with a lower incidence of bronchospasm in high risk patients than a total intravenous anesthetic with propofol and remifentanil [59].

### Steroid

Intravenous steroids should be given early during severe bronchospasm but they will only be effective after several hours. Intravenous methylprednisolone has an onset time of 1-2 hours with a peak effect at 4-6 hours [60]. Other agents need to be relied upon to control the bronchospasm until this time. In children dexamethasone has been shown to be a viable alternative to prednisone [61].

### Intravenous ketamine

Ketamine is powerful bronchodilator drug. Unfortunately, it induces tachycardia and this limits the dose that can be safely administered during severe bronchospasm because tachycardia is a prominent feature of both hypercapnia and beta-2 agonist therapy. At higher doses hallucinations become a major side effect and this drug should be avoided in schizophrenia.

### Inhaled anticholinergics

Inhaled anticholinergic drugs like ipratropium were initially reserved for the treatment of COPD but they are increasingly found to be effective in poorly controlled asthma in both children and adults [62-64].

### Helium

Heliox is a mixture of helium and oxygen and has been used in acute asthma exacerbations. The reduced density of the helium gas improves gas flow under turbulent conditions. Heliox itself does not seem to be a bronchodilator. But it has been recommended as a driving gas for nebulized beta-2 agonists [65]. It enhances drug delivery of inhaled medications by increasing the deposition of drugs in the distal airways [66]. Unlike the other noble gas xenon, helium does not have an anesthetic effect and does not cause sedation [67].

### Methylxanthines

Intravenous methylxanthines like aminophylline are not frequently used anymore for the treatment of acute asthma due to the low therapeutic index of these drugs. Side effects are nausea, vomiting, seizures and serious cardiac arrhythmias. In severe asthma, however, some benefit has been demonstrated. Serum levels were monitored in these studies to prevent toxicity [68,69].

### Leukotriene receptor antagonists

Montelukast, a Leukotriene receptor antagonist, has been used with some success in acute severe asthma [70-73]. Montelukast is more effective when given intravenously than orally [74]. However, the treatment effect is small and in one randomized clinical study in children IV montelukast was found to be ineffective [75].

### Extracorporeal CO_2_ removal

Extracorporeal circuits can be used to eliminate CO_2_ from the blood stream. There is limited systematic evidence evaluating the efficacy of this advanced therapy of last resort [76]. A data analysis from the extra-corporeal life support organization (ELSO) database revealed a survival rate of 84%. Complications like hemorrhage, renal impairment, neurologic sequelae and infection were common however [77]. This potentially dangerous therapy should, therefore, only be employed when all other therapies have failed to achieve the necessary therapeutic effect.

### Epinephrine

It should only be used if there is suspicion of anaphylaxis due to associated clinical features or there is hypotension that cannot be corrected with IV fluids or treatment for dynamic hyperinflation. Beta-2 agonists are preferred to epinephrine because they are less prone to induce tachy-dysrhythmias.

### Deepening of anesthesia

Deepening the level of general anesthesia is often recommended to improve bronchospasm. This should be done with agents that are powerful bronchodilators like dexmedetomidine, lidocaine, sevoflurane and ketamine. Deepening anesthesia with opioids may increase bronchospasm or lead to chest wall rigidity and using propofol is ineffective. Propofol did not alter the contractile response to electric field stimulation in human tracheal rings while remifentanil increased it and lidocaine completely abolished it [78].

Reoccurrence of bronchospasm is a common problem on emergence from general anesthesia because the sevoflurane has been turned off. Additional beta-2 agonist and IV bronchodilators maybe needed to control bronchospasm during emergence. Neostigmine exacerbates bronchospasm [79] and neuromuscular blocking drugs should, therefore, be reversed with sugammadex if the patient is wheezing. Administering an albuterol nebulizer immediately after extubation may be helpful.

## Ventilation during Severe Bronchospasm

Ventilation during severe bronchospasm is usually very difficult. Airway pressures are high and gas trapping in the thorax may occur. The patient is unable to exhale fully prior to the ventilator pumping another tidal volume breath into the lungs. This will lead to the dreaded “dynamic hyperinflation syndrome” [80]. In this clinical entity, there is a progressive rise of intrapulmonary pressure with each breath. Eventually the intrathoracic pressure will be so high that the venous return to the heart is impaired and the patient suffers from systemic hypotension.

### Diagnosis of dynamic hyperinflation

The jugular veins are distended and the chest is hyperinflated. The pulses are weak or impalpable, and the electrocardiogram (ECG) shows a sinus tachycardia without features of myocardial ischemia. If ventilation is continued without making any changes this condition eventually will develop into pulseless electrical activity (previously known as electro-mechanical dissociation), cardiac arrest, and eventually death. The pathophysiology and cardiovascular effects of this condition are very similar to tension pneumothorax, even though no pneumothorax is present. If hypotension develops in a patient with severe bronchospasm, the ventilator circuit should therefore be disconnected from the ETT and the flow of gas at the end of the ETT should be observed. There is usually a prolonged wheeze emanating from the ETT under these circumstances. If the hypotension resolves after disconnecting the patient from the breathing circuit, the diagnosis of dynamic hyperinflation syndrome has been confirmed.

### Treatment of dynamic hyperinflation

After dynamic hyperinflation has been recognized, the clinician should adopt a ventilation strategy of permissive hypercapnia. In this strategy, the goal of positive pressure ventilation is no longer to eliminate CO_2_ from the blood stream and maintain a normal arterial partial pressure of CO_2_ (PaCO_2_). Instead the therapeutic aim is to ensure adequate oxygenation while at the same time lowering intrathoracic pressure below venous return pressure, to allow for adequate venous return to the right heart and the maintenance of an adequate cardiac output. The respiratory rate should be reduced to allow for a longer expiration time [81]. An inspiration to expiration (I:E) ratio of 1:6 or higher will reduce the chance that the patient did not have enough time to fully exhale prior to the ventilator initiating the next inspiration. Larger tidal volumes allow for better exhalation because the diameter of the conducting airways is increased at higher lung volumes (Table 5).

The normal pressure gradient for venous return is only about 4-8 mmHg [82]. An increase in intrathoracic pressure decreases cardiac output [83]. Administration of vasopressors and IV fluid can transiently increase venous return but eventually this will not be able to overcome the raised intrathoracic pressure. Disconnection from the ventilator will then become necessary to allow for adequate venous return and cardiac output. The hyper-carbic respiratory acidosis that results from a ventilation strategy of permissive hypercapnia is very well tolerated by the body [84-87]. The main features of hypercarbia are tachycardia, vasodilation with erythema and an increase in intracerebral pressure. The main contraindication to permissive hypercapnia is therefore the presence of acutely raised intracranial pressure. The vasodilation from the hypercarbia is usually mild and readily responds to vasopressor drugs. The permissive hypercapnia strategy will need to be maintained until the bronchospasm has improved enough to allow for adequate venous return. Depending on the clinical circumstances this may take several hours. Once the bronchospasm has been controlled normal ventilation with conventional minute volume and PaCO_2_ targets can be re-instituted.

In summary, obstruction of airflow and bronchospasm are seen with increasing frequency in the perioperative period. Non-traditional treatment modalities like IV dexmedetomidine, lidocaine, magnesium, ketamine, steroids and inhaled sevoflurane are very effective but currently underused. Just like with pain therapy, the treatment of acute severe airflow obstruction needs to be multimodal to achieve maximal bronchodilation while limiting the side effects of each of the individual therapy. Combining these agents with first line beta-2 agonist therapy, will result in better control of airflow obstruction with fewer side effects. When hypotension is presented, both anaphylaxis and the dynamic hyperinflation of the lungs needs to be excluded. If hyperinflation is present, a ventilation strategy of permissive hypercapnia should be adopted until the bronchospasm is under control. When all the modalities of treating bronchospasm are employed in combination and in a timely fashion, CO_2_ removal via an extra-corporeal circuit is usually not necessary.

## Figures and Tables

**Table 1:** Differential diagnosis of bronchospasm.

Heart failure
Mass compressing intrathoracic airways
Aspiration
Kinked endotracheal tube
Anaphylaxis
Pulmonary edema due to fluid overload

**Table 2:** Things to avoid in patients with bronchospasm.

Inhalation of cold dry gases
Endotracheal intubation
Bronchial suctioning
Bronchoalveolar lavage
Rigid or flexible bronchoscopy

**Table 3:** Drugs to avoid in patients at risk of bronchospasm.

Vancomycin
Morphine
Succinylcholine
Racemic atracurium (cis-atracurium is fine)
Mivacurium
Sodium pentothal
Desflurane
Ketorolac and other non-steroidal anti-inflammatory drugs
Non-selective beta blockers like labetalol or propranolol
Iodine based contrast agents

**Table 4:** Drugs in the treatment of perioperative bronchospasm.

Beta-2 agonists (albuterol)
Dexmedetomidine
Lidocaine
Magnesium
Sevoflurane
Steroids
Ketamine
Anti-cholinergics (ipratropium)
Helium
Methylxanthines (aminophylline)

**Table 5:** Treatment of dynamic hyperinflation syndrome.

Disconnect the patient from the ventilator until pulses return
Adopt a ventilation strategy of permissive hypercapnia
Low respiratory rate to increase time for expiration
Large tidal volume to increase airway diameter
Maintain this strategy until bronchospasm has been controlled
